# Effects of 4-hexylresorcinol administration on the submandibular glands in a growing rat model

**DOI:** 10.1186/s13005-022-00320-7

**Published:** 2022-06-06

**Authors:** Joo-Hyung Yoon, Dae-Won Kim, Suk Keun Lee, Seong-Gon Kim

**Affiliations:** 1 Department of Oral and Maxillofacial Surgery, College of Dentistry, Gangneung-Wonju National University, Jukheon gil 7, 25457 Gangneung, Gangwondo Republic of Korea; 2 Department of Oral Biochemistry, College of Dentistry, Gangneung-Wonju National University, 28644 Gangneung, Korea; 3 Institution of Hydrogen Magnetic Reaction Gene Regulation, 34140 Daejeon, Korea

**Keywords:** 4-hexylresorcinol, Salivary gland, Angiogenesis, Testosterone

## Abstract

**Background:**

4-Hexylresorcinol (4HR) is a food additive and class I histone deacetylase inhibitor. In this study, we examined the effects of 4HR administration on the submandibular gland in a growing rat model.

**Methods:**

Four-week-old rats were used in this study. The experimental group (nine males and eight females) received 12.8 mg/kg of 4HR weekly for 12 weeks. Ten rats (five males and five females) were used as controls. The submandibular glands of rats were collected 12 weeks after the first administration of 4HR. The weight of the glands was measured. Histological analysis, immunoprecipitation-high-performance liquid chromatography (IP-HPLC), and western blotting were performed.

**Results:**

The weights of the rat submandibular glands were higher in the experimental groups than in the control group, especially in male rats (*P* < 0.05). The vascular endothelial growth factor (VEGF) and testosterone in the submandibular glands were more highly expressed in 4HR-treated male rats than in untreated rats, as detected by both western blotting and immunohistochemistry. The IP-HPLC results demonstrated that the expression levels of Ki67, epidermal growth factor, and testosterone in the submandibular glands were higher in 4HR-treated male rats than in untreated rats.

**Conclusions:**

This study demonstrated that the systemic administration of 4HR increased the weight of submandibular glands in male rats. In addition, the testosterone and VEGF expression levels in the submandibular glands increased owing to 4HR administration.

## Background

4-Hexylresorcinol (4HR) is a synthetic alkyl resorcinol [1]. Recently, it was discovered that 4HR functions as a histone deacetylase inhibitor (HDACi) [2]. Histone deacetylase (HDAC) is an enzyme that removes acetyl groups that neutralize the positive charge of histones [3]. The removal of acetyl groups enhances the combination of histones and DNA, leading to the suppression of DNA translation [4]. HDACis are small compounds that inhibit the activity of HDACs and upregulate DNA translation [3].

To the best of our knowledge, there have been no reports on the relationship between HDACi administration and salivary gland physiology. As 4HR is a food additive, it can be ingested by children. Trichostatin A is an HDACi that stimulates the bone morphogenic protein-7 (BMP7) pathway [5]. BMP7 represses the transforming growth factor-β (TGF-β) pathway, which is involved in renal fibrosis [5]. The overexpression of TGF-β is associated with salivary gland fibrosis [6, 7]. Based on these observations, we conclude that the application of 4HR decreases the expression of TGF-β. However, the administration of 4HR increases the expression of TGF-β1, which leads to an increase in angiogenesis [8]. An increase in angiogenesis may lead to an increase in the salivary gland size. 4HR upregulates the expression of bone morphogenic protein-4 (BMP4) in osteoblast-like cells [9]. Elevated BMP4 expression in the salivary glands is associated with the suppression of ductal branching [10]. The administration of 4HR may increase the salivary gland size. However, there have been no reports on the relationship between 4HR application and salivary gland physiology.

According to a recent study [1], the administration of 4HR has different effects on growing male and female rats. Male rats showed reduced mandibular size, whereas female rats showed increased mandibular size after 4HR administration. The administration of 4HR decreases serum testosterone levels in male rats, but increases serum growth hormone levels in both sexes [1]. Hormone levels in the blood may influence salivary gland growth. Unlike humans, submandibular glands are the main salivary glands responsible for the production of biologically active factors in rats [11]. Thus, investigating submandibular glands after 4HR administration is important to understand the 4HR-induced changes in the salivary glands of rats. Hormones and cytokines secreted by salivary glands are important for the homeostasis of the oral mucosa in both humans and rodents [12, 13].

Daily voluntary exercise increases drug-induced saliva secretion in rat submandibular glands by increasing the vascular endothelial growth factor (VEGF) [14]. Increased salivation in rodents and humans is associated with VEGF-induced angiogenesis [15]. The preservation of VEGF expression is important to mitigate diabetic-induced salivary gland microvascular injury [16]. Ki-67 has been used as a marker of proliferation rate in salivary tumors [17]. In rats, Ki-67 immunoreactivity has been observed in the secretory acini and the walls of blood vessels [18]. Therefore, the expression levels of VEGF and Ki-67 in the salivary glands may be useful indicators for the evaluation of functional hypertrophy.

In a previous study, 12.8 mg/kg of 4HR was administered weekly for 12 weeks in growing rats [1]. This dosage of 4HR for systemic administration was selected among the dosages used in a toxicology study performed by the National Health Institute of the United States [19]. The weekly administration of 0.128 mg/kg and 12.8 mg/kg of 4HR, showed that the latter dose accelerates the eruption rate of incisors in rats [20]. The salivary glands in the submandibular space were prepared and donated for this experiment. This study aimed to evaluate the effects of 4HR administration on the submandibular glands of growing rats. Submandibular gland weights were measured. In addition, the expression levels of VEGF and testosterone in the submandibular glands were evaluated.

## Methods

### Sample collection

The tests in this study were performed using donated samples. This study was approved by the Institutional Animal Care and Use Committee of Gangneung-Wonju National University (GWNU-2021-2-1). The detailed procedure for 4HR administration has been described in a previous publication [1]. Briefly, 30 Sprague-Dawley rats (15 males and 15 females) were used in this study. For 12 weeks, 12.8 mg/kg/week of 4HR was injected subcutaneously into 20 rats (10 males and 10 females). Ten rats (five males and five females) served as the control group. Twelve weeks after the first 4HR administration, all rats were euthanized. Three rats (one male and two females) from the 4HR group died before being euthanized and their data were excluded from the analyses.

The salivary glands in the submandibular space were enucleated independently. Both the submandibular and sublingual glands were in the same space in the rats. Two pairs of submandibular and sublingual glands were harvested from each rat. The weight of one of these pairs of glands was measured and the glands were stored for protein analysis, namely immunoprecipitation-high-performance liquid chromatography (IP-HPLC) and western blotting. The sublingual glands were then dissected and discarded. The other specimen was fixed in formalin without dissection. The glands were then used for hematoxylin and eosin (H&E) staining, periodic acid-Schiff (PAS) staining, and immunohistochemistry.

### H&E and PAS staining

For H&E staining, deparaffinization was performed with xylene and rehydration was performed with 100%, 95%, and 70% ethanol. The slides were rinsed with distilled water, and Harris hematoxylin was applied. After being rinsed with tap water, the specimens were decolorized with acid alcohol. After rinsing, the specimens were counterstained with eosin. The specimens were dehydrated using 95% and 100% ethanol and cleared using xylene. Mounting was performed after each process.

The deparaffinization and hydration processes for PAS staining were in accordance with those for H&E staining. The specimens were immersed in a periodic acid solution and rinsed with distilled water. The samples were then immersed in Schiff’s solution and rinsed with distilled water. Next, we performed staining in Mayer’s hematoxylin and the sections were rinsed with distilled water. Dehydration, clearing, and mounting were performed as described for H&E staining.

### Immunohistochemistry

Deparaffinization, rehydration, and washing with distilled water were performed using the same methods as those used for H&E staining. Trypsin tablets were mixed with 1 mL of distilled water. For antigen retrieval, a trypsin solution was added to the slides. After 10 min, the slides were washed with phosphate-buffered saline (PBS). Hydrogen peroxide was used to block endogenous peroxidase activity. The slides were then washed twice with PBS. The blocking solution was applied to the slides for 1 h. All antibodies were purchased from Santa Cruz Biotechnology (Dallas, TX, USA). Primary antibodies against VEGF (CAT#: sc-57,496), testosterone (CAT#: sc-73,144), or Ki-67 (CAT#: sc-23,900) were applied to the slides, which were then covered with paraffin films. The slides were placed in a humid chamber overnight at 4 °C. The next day, the slides were washed thrice with PBS. Secondary antibodies with horseradish peroxidase were applied to the slides, which were then stored in a humid chamber for 15 min. The slides were then washed thrice with PBS. For colorization, a 3,3’-diaminobenzidine (DAB) solution was applied to the slides. After being washed with distilled water, the slides were mounted.

### Western blotting

Proteins were collected from the salivary glands by impact pulverization in liquid nitrogen and the fine fragments were transferred to ice-cold RIPA buffer (Sigma Aldrich, USA) and centrifuged at 12,000 × *g* for 20 min at 4 °C. The protein concentration was measured using the Bradford assay (Bio-Rad, USA). After applying the proteins mixed with a loading buffer (25 mM Tris, 0.1% SDS, and 0.2 M glycine), gel electrophoresis was performed. The proteins in the gel were transferred onto a nitrocellulose membrane. Blocking was performed using 5% non-fat dry milk. Primary and horseradish peroxidase-conjugated secondary antibodies were used. Antibodies against VEGF (CAT#: sc-57,496) and testosterone (CAT#: sc-73,144) were used as the primary antibodies. The protein bands were detected using an enhanced chemiluminescence system (Amersham Pharmacia Biotech, Piscataway, NJ, USA). The bands were digitally imaged using a ChemiDoc XRS system (Bio-Rad Laboratories, Hercules, CA, USA). β-actin was used as a control [21].

### Immunoprecipitation high-performance liquid chromatography (IP-HPLC)

Tissue proteins were obtained from the extracted submandibular glands of 16-week-old rats treated with 4HR (12.8 mg/kg/week for 12 weeks), and each extracted protein was immunoprecipitated using antisera against Ki-67, epidermal growth factor (EGF), insulin-like growth factor-1 (IGF-1), estrogen receptor-β (ERβ), TGF-β1, growth hormone (GH), testosterone, α-smooth muscle actin (α-SMA), and IgA, followed by HPLC analysis. Proportional data (%) were plotted on a line graph and a star plot. The expression of housekeeping proteins, such as β-actin, was compared with that of the untreated control (≤ 5%).

### Statistical analysis

The mean value ± standard deviation was calculated for each group. Statistical analyses were performed using SPSS version 12 (SPSS Inc., Chicago, IL, USA). Equality of variance was examined using Levene’s test. Comparisons between groups were performed using the independent samples *t*-test. Significance was evaluated using a two-tailed test. The level of significance was set at *P* < 0.05.

## Results

**Fig. 1:**
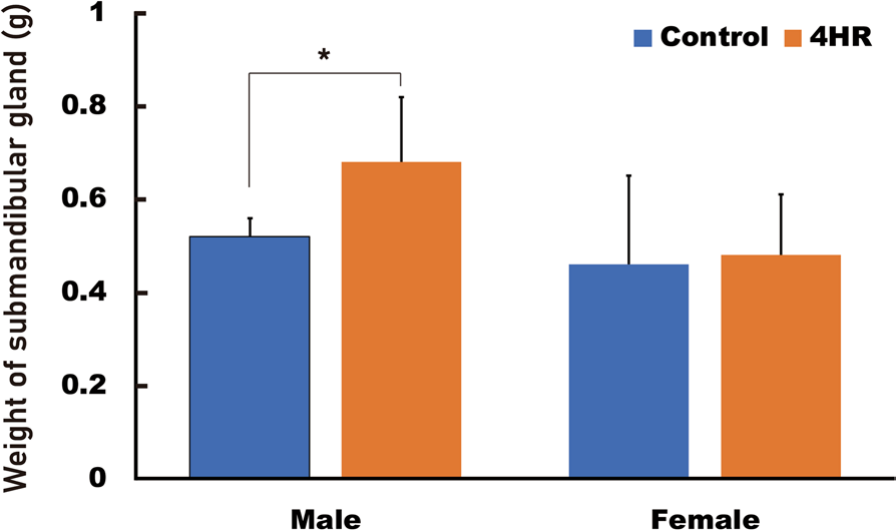
The weight of the submandibular glands and histological findings. The weight of the submandibular glands in the 4HR group was significantly higher than that of the untreated control in males (*P = 0.036)

**Fig. 2:**
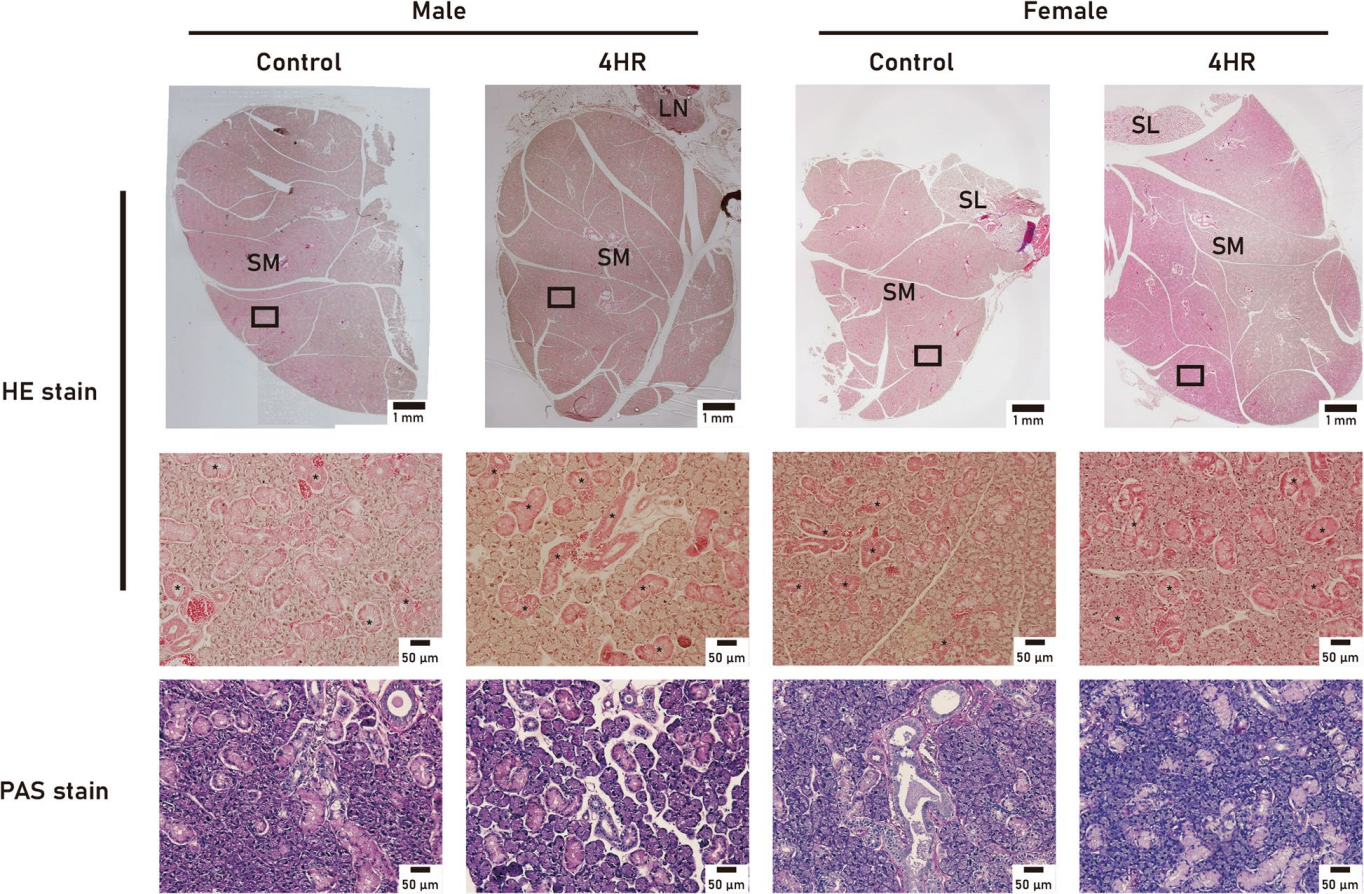
Histological findings of submandibular glands. Hematoxylin and eosin (H&E) stain and periodic acid-Schiff (PAS) stain. The low magnification view shows the submandibular gland (SM), the sublingual gland (SL), and lymph node (LN). The convoluted ducts (*) were well developed in the gland. There was no significant difference in the structure and size of the cells or the amount of fibrous tissue between the groups

The submandibular glands were dissected and their weights were measured and compared. The mean weight of the submandibular glands in the 4HR group was higher than that of the untreated control in both female and male rats (Fig. 1). However, there were no significant differences between female rats (*P* > 0.05). In the male rats, the mean weight of the submandibular glands in the 4HR group was 0.68 ± 0.14 g and that in the untreated control was 0.52 ± 0.04 g. The weight of the submandibular glands in the 4HR group was approximately 19% higher than that in the untreated control group (*P* = 0.036). Histological examination using H&E and PAS staining was performed to investigate the reason for the weight difference (Fig. 2). However, there were no significant differences in the structure and size of the cells or the amount of fibrous tissue between the groups.

**Fig. 3:**
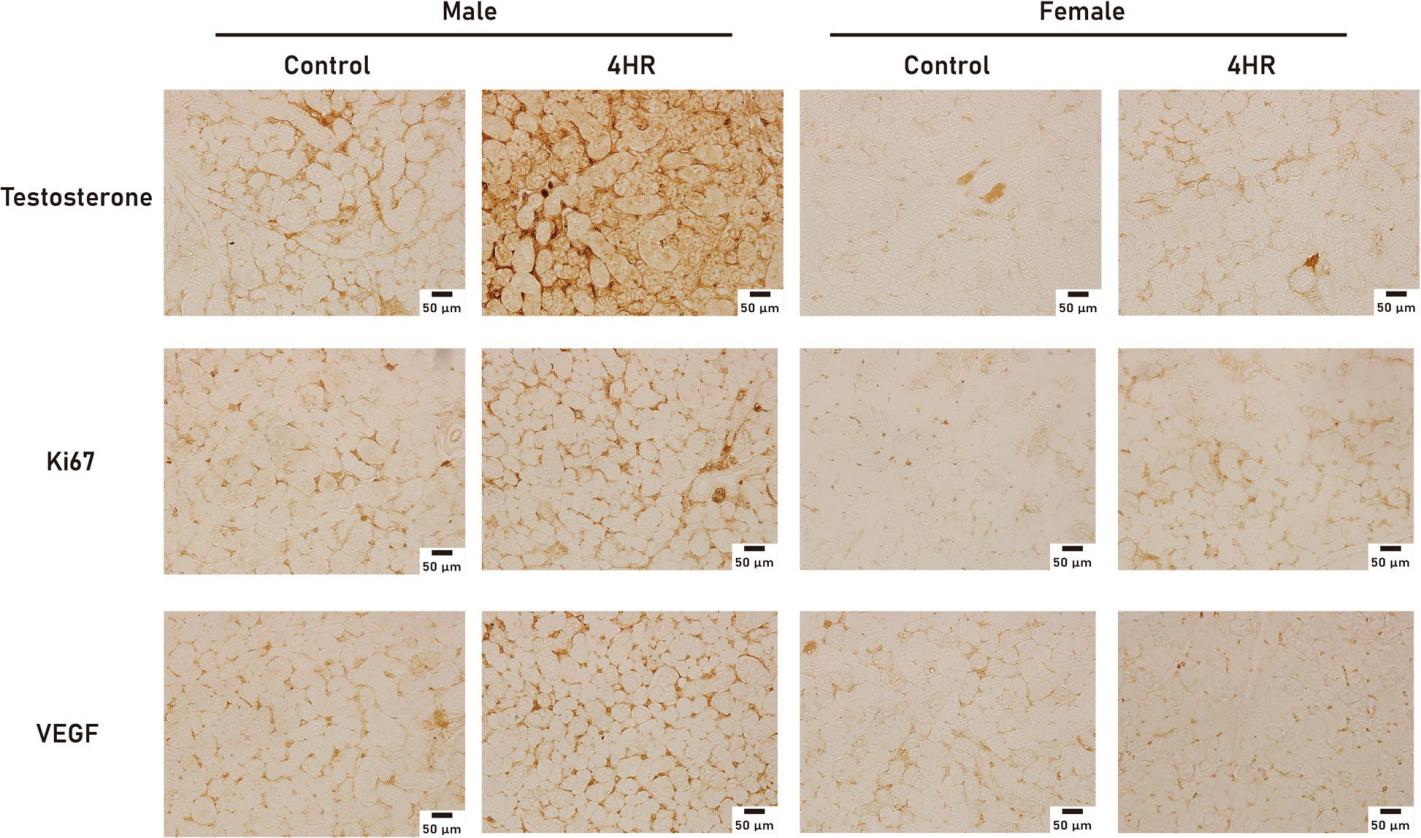
Immunostaining for VEGF, testosterone, and Ki67. The staining intensity was higher in the 4HR group than in the untreated control group

**Fig. 4:**
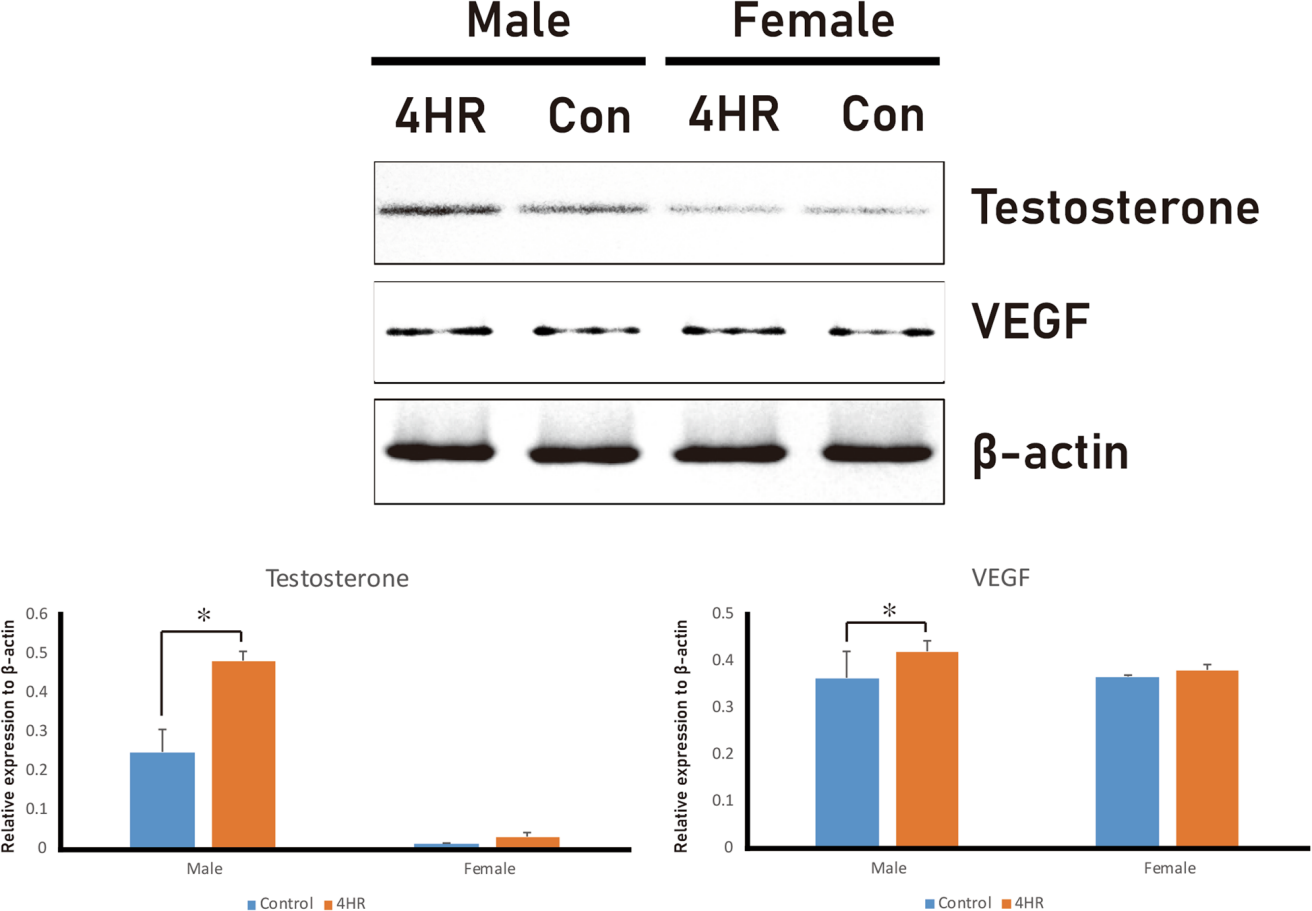
Western blotting for VEGF and testosterone. The expression levels of both proteins were significantly higher in the 4HR group than in the untreated control

**Fig. 5:**
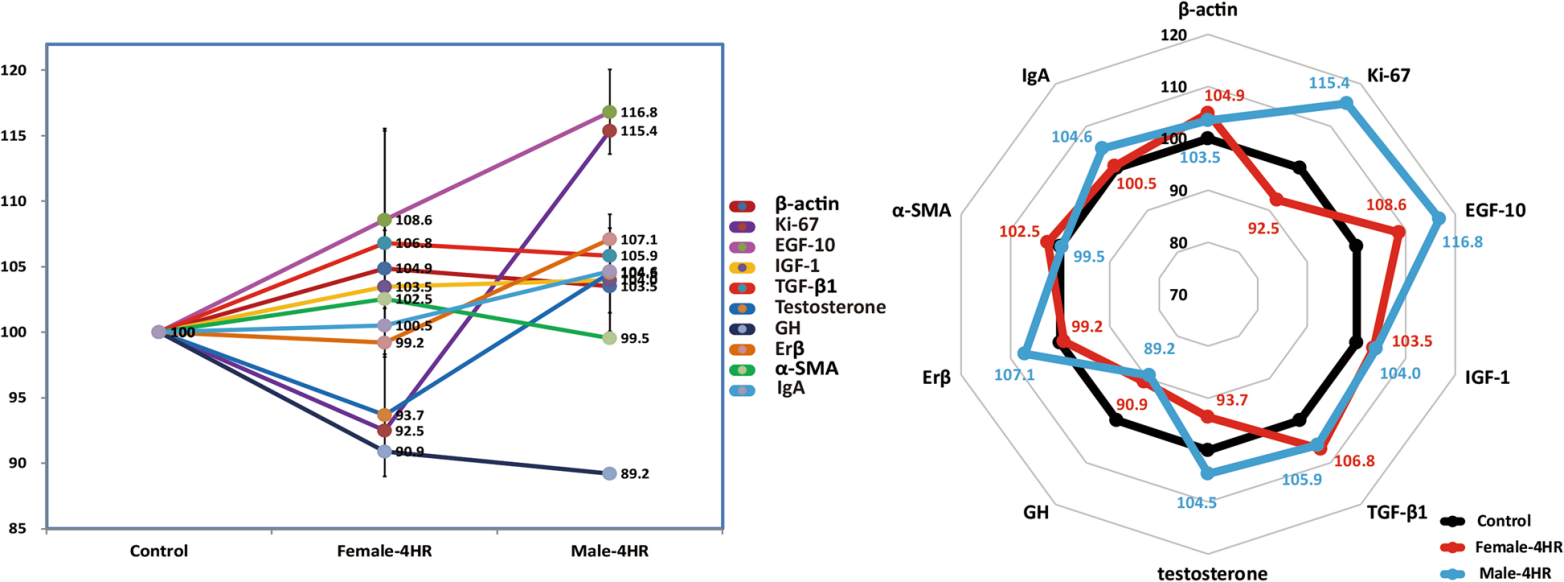
IP-HPLC results. 4HR-treated male rats showed increased expression levels of Ki-67, epidermal growth factor (EGF), insulin-like growth factor-1 (IGF-1), transforming growth factor-β1 (TGF-β1), testosterone, and estrogen receptor-β (ERβ) compared to untreated male rats. 4HR-treated female rats showed decreased levels of growth hormone, Ki-67, and testosterone compared to untreated female rats

Angiogenesis and hormonal effects play an important role in gland hypertrophy. Upon immunohistochemistry with antibodies for VEGF, testosterone, and Ki67, staining was more intense in the 4HR group than in the untreated control group (Fig. 3). According to the western blotting results, both the VEGF and testosterone levels were significantly higher in the 4HR group than in the untreated control group (Fig. 4). The protein expression levels in salivary glands were determined using IP-HPLC (Fig. 5). The IP-HPLC results showed that 4HR-treated male rats had significantly increased levels of Ki-67 (15.4%), EGF (15.4%), IGF-1 (4.0%), TGF-β1 (5.9%), testosterone (4.5%), and ERβ (7.1%) compared to untreated male rats, whereas 4HR-treated female rats showed decreased levels of growth hormone (GH, 9.1%), Ki-67 (7.5%), and testosterone (6.3%) compared to untreated female rats.

## Discussion

In this study, the mean weight of the submandibular glands was higher in the 4HR treated male group than in the untreated male control group (Fig. 1). The microstructure of the salivary glands was examined using H&E and PAS staining, and there was no gross difference between the groups (Fig. 2). The expression levels of VEGF, testosterone, and Ki-67 increased in the submandibular glands of 4HR-treated male rats compared to those in untreated male rats (Figs. 3 and 4). To the best of our knowledge, this is the first report on the effect of 4HR administration on the submandibular glands of growing rats.

Four hours is a type of alkyl resorcinol [1]. Alkyl resorcinols have been used as food additives, oral gargling products, and cosmetics [22–24]. As alkyl resorcinols have several beneficial properties, such as antioxidant [25], anticancer [25, 26], and neuroprotective ones [25], while they can also prevent fatty liver [25] and improve pancreatic beta cell function [25], their applications are expected to expand. However, their potential effect on salivary glands has not yet been studied. To expand the indications for alkyl resorcinol, the effects of its administration on salivary gland function should be studied. 4HR is a widely used alkyl resorcinol in the food [1, 23], cosmetics [27], and medical industries [1]. According to a previous study [1], 4HR administration in growing male rats decreased serum testosterone levels. The reduced production of steroid hormones is a frequent finding in the application of alkyl resorcinols [25]. The decrease of the synthesis of steroid hormones by alkyl resorcinol may be responsible for the decreased serum testosterone levels. However, in this study, the testosterone levels in the submandibular glands of 4HR-treated male rats increased (Figs. 3 and 4), despite a reduction in serum levels [1].

In this study, increased salivary testosterone levels in male rats were confirmed by immunohistochemistry (Fig. 3), western blotting (Fig. 4), and IP-HPLC (Fig. 5). Although 4HR-treated male rats exhibited lower serum testosterone values than untreated male rats, the values in both groups were within the physiological range [1]. According to a study by Ieko et al. [28], testosterone is synthesized in rat salivary glands, and the amount of testosterone produced in the salivary glands is similar to that produced in the testes. Based on the results of this study (Figs. 3 and 4) and facts reported in a previous study [28], it could be inferred that the decreased synthesis of testosterone in the testes after the application of 4HR induced an increase in the testosterone synthesis in the salivary glands as a compensatory mechanism. In addition, increased testosterone levels could lead to increased EGF expression (Fig. 5), which is related to the growth of salivary glands [29, 30]. In this study, the EGF levels increased significantly owing to 4HR administration (*P* < 0.05; Fig. 5). Ki-67 is a proliferation marker and its expression was significantly higher in male rats treated with 4HR (Figs. 3 and 5). Hence, it can be inferred that the significant weight increase in the submandibular glands obtained from the male experimental group (Fig. 1) may be due to the increase in EGF and Ki-67 expression induced by salivary testosterone (Figs. 3 and 4, and 5). Based on the findings of the current study, the decrease in serum testosterone levels observed in a previous study [1] might be due to the suppressive effect of 4HR on the testes. Drugs with anticancer properties usually inhibit testicular function [31, 32]. The administration of 4HR inhibits tumor proliferation [33]. In contrast to the testes, normal salivary glands do not exhibit high mitosis. Accordingly, salivary glands may produce more testosterone to compensate for the reduced testosterone production in the testes after 4HR administration. However, the production patterns of sex hormones are highly divergent among species [34–37]. Further studies are needed to confirm this hypothesis.

The administration of 4HR increases the expression levels of VEGF and TGF-β1 in human umbilical vein endothelial cells [21]. In this study, the expression levels of VEGF and TGF-β1 were higher in 4HR-treated rats (Figs. 4 and 5). 4HR enhances the expression of TGF-β1, and by the downstream signal in the RAS/SMAD pathway, which begins after TGF-β1 binds to ALK5, vascular regeneration is stimulated [8]. Based on the immunohistochemistry results of this study (Fig. 3), it can be inferred that vascular regeneration stimulated by the RAS/SMAD pathway occurred in the submandibular glands after the administration of 4HR. Angiogenesis is a vital step in the production of cytokines and hormones in the salivary glands. Therefore, increased VEGF and TGF-β1 expression induced by the 4HR treatment may contribute to increased glandular size and testosterone production. VEGF is primarily produced by the parotid glands in humans, but is also produced by the submandibular glands in rats [11]. The expression level of VEGF in salivary glands does not change dramatically with age [38]. The expression level of VEGF in salivary glands is sufficiently high to induce angiogenesis in salivary glands in an autocrine manner [39]. Increased VEGF levels may increase the production of hormones by increasing the blood supply to cells in salivary glands [40]. Increased VEGF and testosterone levels were observed in the submandibular glands of 4HR-treated male rats (Figs. 3 and 4).

Gland hyperplasia after 4HR administration was not the first finding. Benign hyperplasia of the adrenal gland has been observed in male rats two years after the oral administration of 4HR [41]. The histological structure of the hyperplastic adrenal gland after 4HR administration does not differ from that of the normal adrenal gland [42]. As alkyl resorcinol suppresses steroid production [25], the hyperplasia of the adrenal gland after 4HR administration is poorly understood. In this study, the weight of the submandibular glands was higher in male rats treated with 4HR than in untreated controls (Fig. 1). Similar to the effects observed in the adrenal gland, this change may result from reactive hyperplasia in response to reduced serum testosterone levels.

This study has several limitations. The tests in this study were performed using donated samples that were enucleated in a previous study [1]. Therefore, unnecessary animal euthanasia was avoided. However, the examination of the testes would be helpful in understanding the underlying mechanisms. Thus, the next study should be performed on testes. In addition, there are large differences between the salivary glands of rats and humans. In humans, the parotid gland is the main gland involved in the production of growth factor [43]. The saliva from the human parotid gland contains testosterone [44]. However, it is unclear whether testosterone originates from the blood or salivary glands. As the serum level of testosterone is significantly lower in 4HR-treated male rats [1], increased testosterone levels in the submandibular gland of the same animals may be due to the production of the submandibular gland itself.

In conclusion, it can be inferred that 4HR induces the hypertrophy of submandibular glands in males and increases testosterone, VEGF, and Ki67 production. Considering the reduced serum testosterone levels in the same animal model [1], these changes in the submandibular gland might be a compensatory phenomenon for the reduced serum testosterone levels induced by 4HR administration.

## Data Availability

Data sharing is not applicable to this article because no datasets were generated or analyzed during the current study.

## Abbreviations

4HR: 4-hexylresorcinol
HDAC: histone deacetylase
HDACi: histone deacetylase inhibitor
BMP7: bone morphogenic protein-7
TGF-β: transforming growth factor-β
PAS: periodic acid Schiff
EGF: epidermal growth factor
